# *Sanguisorba officinalis* L. suppresses non-small cell lung cancer *via* downregulating the PI3K/AKT/mTOR signaling pathway based on network pharmacology and experimental investigation

**DOI:** 10.3389/fphar.2022.1054803

**Published:** 2022-11-24

**Authors:** Hong Li, Jing Lin, Fei Yang, Junzhu Deng, Jia Lai, Jing Zeng, Wenjun Zou, Nan Jiang, Qianqian Huang, Hua Li, Jian Liu, Mao Li, Zhirong Zhong, Jianming Wu

**Affiliations:** ^1^ School of Pharmacy, Southwest Medical University, Luzhou, China; ^2^ Laboratory of Ethnopharmacology, Tissue-orientated Property of Chinese Medicine Key Laboratory of Sichuan Province, West China School of Medicine, West China Hospital, Sichuan University, Chengdu, China; ^3^ School of Pharmacy, Chengdu University of Traditional Chinese Medicine, Chengdu, China; ^4^ The Key Laboratory of Medical Electrophysiology, Ministry of Education of China, Institute of Cardiovascular Research, Luzhou, China; ^5^ School of Basic Medical University, Southwest Medical University, Luzhou, China

**Keywords:** *Sanguisorba officinalis* L., non-small cell lung cancer, network pharmacology, PI3K/AKT/mTOR, ROS

## Abstract

**Background:** Non-small cell lung cancer (NSCLC) is the most common type of lung cancer. *Sanguisorba officinalis* L*.* (SOL), a traditional Chinese herbal medicine called Diyu, has been shown to have potent antitumor effects. However, the role of SOL in suppressing NSCLC remains unknown.

**Methods:** Network pharmacology was employed for acquiring the potential targets and mechanisms of SOL in NSCLC. Based on the predictions of network pharmacology, we used CCK8 and EdU assays to investigate cell proliferation, flow cytometry to investigate apoptosis, wound healing assay to investigate cell migration, and transwell assay to investigate cell invasion *in vitro*. Western blot was employed for detecting the potential proteins, including signaling pathways and apoptosis. The A549-bearing athymic nude mice were employed to verify the effect on cell proliferation and apoptosis *in vivo*.

**Results:** SOL significantly inhibited the proliferation, migration and invasion of NSCLC cells in a dose-dependent manner. Flow cytometry showed that the apoptotic ratio and ROS level of NSCLC cells increased significantly with increasing concentrations. AKT and the PI3K-AKT signaling pathway were analyzed as the most relevant target and pathway *via* network pharmacology predictions. Western blotting revealed that the expression levels of p-PI3K, p-AKT, and p-mTOR in NSCLC cells treated with SOL were significantly downregulated, while cleaved PARP-1 and caspase-3 were upregulated in a dose-dependent manner. The results in the mouse xenograft model were consistent with those in NSCLC cell lines.

**Conclusion:** SOL downregulated the PI3K/AKT/mTOR signaling pathway to suppress NSCLC.

## 1 Introduction

Lung cancer has the highest mortality rate among all cancers according to the Global Cancer Case Report 2020. Lung cancer can be divided into small cell lung cancer and non-small cell lung cancer (NSCLC). NSCLC is accounting for 80%–85% of all lung cancers and has low five-year survival rates (Planchard et al., 2018; Sung et al., 2021). Currently, chemotherapy, immunotherapy, radiotherapy, and surgery are the main therapeutic options for NSCLC. Among these, chemotherapy plays a critical role in the treatment of all stages of NSCLC. Traditional medicines harm various normal cells, while targeted drugs (e.g., EGFR TKIs, ALK TKIs, ROS1 TKI, and NTRK TKI) are susceptible to resistance (Chen et al., 2020; Rodak et al., 2021). Therefore, breakthroughs in lung cancer chemotherapy are urgently needed.

Searching for potential antitumor-active components from traditional Chinese medicine (TCM) has become the focus of pharmaceutical research. Current studies have shown that TCMs, including *Cremastra appendiculata* (Wang et al., 2021), *Artemisia Annua* (Zhang S. Q et al., 2020) and *Rhodiola rosea* L. (Zhang S. Q et al., 2020), can be used as candidate drugs against NSCLC. *Sanguisorba officinalis* L. (SOL) is a medicinal plant widely distributed in China (Zhou et al., 2021). Currently, “Diyu Sheng Bai Pian,” made from SOL, is used clinically to treat leukopenia induced by antitumor drugs (Zhao, 2019). Many studies have shown that SOL can markedly inhibit the growth of some solid tumors, such as colorectal cancer, prostate cancer, and breast cancer (Choi et al., 2012; Liu et al., 2016; Wang N et al., 2020). We previously demonstrated that 3,3′,4′-trimethylellagic acid, isolated from SOL, could markedly inhibit colon cancer (Bai et al., 2019) and regulate platelet levels (Li et al., 2021). And the ethanol extract of SOL could induce apoptosis in hepatocellular carcinoma cells by inhibiting the phosphoinositide 3-kinase (PI3K)/AKT pathway (Jiang et al., 2021). These studies highlight the significant antitumor effects of SOL. Thus, it will be interesting to explore the antitumor effects and significance of SOL in non-small cell lung cancer.

Network pharmacology, based on systems biology and network analysis theory, is a novel and unique method for systematically studying and exploring the complex relationship between drugs and diseases. It can reveal the complex mechanisms of TCM in the treatment of diseases by constructing and visualizing the drug-disease-target interaction network (Poornima et al., 2016; Wang L et al., 2020). It can effectively map the unexplored target space for natural products, thus providing a systematic approach to expand the drug space, involving proteins in a variety of complex diseases (Kibble et al., 2015; Oettrich et al., 2016). Network pharmacology guides drug research and has an important reference value for the study of drug mechanisms. Therefore, it is necessary to predict and verify the main targets and pathways of SOL activity in lung cancer cells using this approach.

In this study, multiple databases and computational tools were used to establish a pharmacological network to explore potential drug targets and molecular mechanisms of SOL in NSCLC. We have successfully predicted that the active components in SOL can influence NSCLC by PI3K/AKT/mTOR pathway. Various experiments, including cell proliferation, apoptosis, migration, and invasion, further verified the efficacy and mechanism of NSCLC cell lines and mouse xenograft models. Collectively, SOL downregulated the PI3K/AKT/mTOR signaling pathway to suppress NSCLC.

## 2 Materials and methods

### 2.1 Predicting putative *Sanguisorba officinalis* L*.* targets

The chemical composition of SOL was obtained from the Traditional Chinese Medicine Systems Pharmacology Database (TCMSP; http://lsp.nwu.edu.cn/tcmsp.php) (Ru et al., 2014), the Traditional Chinese Medicine Integrated Database (TCMID; http://www.megabionet.org/tcmid/) (Huang et al., 2018), and the Encyclopedia of Traditional Chinese Medicine (ETCM; http://www.tcmip.cn/ETCM/) (Xu et al., 2019). Bioactive compounds were selected according to the following criteria: oral bioavailability (OB) > 30% and drug-likeness (DL) > 0.18. Molecular structures of the selected bioactive compounds were acquired from the PubChem Compound Database (https://www.ncbi.nlm.nih.gov/pccompound) and uploaded to the Swiss Target Prediction Database (http://www.swisstargetprediction.ch/) to obtain putative targets.

### 2.2 Identifying the potential targets of *Sanguisorba officinalis* L*.* activity in NSCLC

The GeneCards database (https://www.genecards.org/), DisGeNET database (http://www.disgenet.org/), and OMIM database (https://www.OMIM.org/) were searched to identify targets related to NSCLC. The targets of both SOL and NSCLC were considered potential therapeutic targets (i.e.,: common targets of SOL and NSCLC) for SOL activity in NSCLC.

### 2.3 Network construction

#### 2.3.1 Construction of drug-disease-target network

Based on the target information of SOL and NSCLC, Cytoscape version 3.7.1 software was employed to construct the network among the bioactive compounds of SOL, NSCLC, and their common targets.

#### 2.3.2 Construction of a protein-protein interaction network and screening of the core targets of *Sanguisorba officinalis* L*.* activity in NSCLC

The interaction network of the bioactive compound targets in NSCLC was built using STRING version 11.0 software (http://string-db.org). Protein interactions with confidence scores greater than 0.4 were analyzed (Szklarczyk et al., 2017). Nodes and score information was imported into Cytoscape 3.7.1 for visual analysis. Topological analysis was conducted by network analysis in Cytoscape 3.7.1, and the core targets were screened according to the main parameters, degree, betweenness centrality, and closeness centrality. Nodes with topological parameters greater than the medians of betweenness centrality and closeness centrality, and greater than twice the degree median, were selected as the core targets (Wang L et al., 2020). The interaction network of core targets was constructed using Cytoscape 3.7.1.

#### 2.3.3 Gene ontology and pathway enrichment analysis of core targets

The Database for Annotation, Visualization, and Integrated Discovery (DAVID; https://david.ncifcrf.gov/) was employed to perform GO function enrichment analysis and KEGG pathway enrichment analysis of core targets. (Huang da et al., 2009). Results were visualized using the Omicshare cloud platform (http://www.omicshare.com/).

### 2.4 Preparation of *Sanguisorba officinalis* L*.* ethanol extracts

As previously reported (Jiang et al., 2021), dried SOL roots were crushed into a crude powder, which was then extracted by sonication in 70% ethanol for 30 min. The resulting solution was filtered and concentrated under a reduced-pressure condenser to acquire the dried ethanol extract of SOL.

### 2.5 UPLC-MS analysis of *Sanguisorba officinalis* L*.* compounds

SOL compounds were analyzed using a UPLC (Exion)–quadrupole time-of-flight (QTOF) MS system (X500R; SCIEX, MA, United States). A Kinetex C18 column (100 mm × 2.1 mm, 2.6 μm, 100 Å; Phenomenex) was used for chromatographic separation. The column temperature was set to 40°C. Mobile phase A was 0.1% formic acid-water (v/v), while mobile phase B was 0.1% formic acid-acetonitrile (v/v). The flow rate was set at 0.3 ml/min. The gradient elution protocol was as follows: 5% B from 0–2.00 min, 5%–70% B from 2.01–18.00 min, and 70%–100% A from 18.0–20.00 min. The injection volume was 5 µl.

### 2.6 Cell culture

NSCLC cell lines (A549 and H1299) obtained from the American Type Culture Collection (Manassa, VA, United States) were used for *in vitro* experiments. Cells were cultured in RPMI 1640 media (#8119060, Gibco, MA, United States) supplemented with 10% fetal bovine serum (FBS, #42Q9281K, Gibco, MA, United States) and 100 U/ml penicillin-streptomycin (#C0222, Beyotime, Shanghai, China), and maintained at 37°C, 5% CO_2_ in a humidified incubator.

### 2.7 Cell viability

A549 and H1299 cells were seeded into 96-well plates (4 × 10^3^ cells/well) and incubated for 24 h before treatment with different concentrations of SOL (0, 15.625, 31.25, 62.5, 125, 250, 500, or 1,000 μg/ml) for 24 h and 48 h 293T, LO2, and Beas-2b cells were seeded into 96-well plates and incubated for 24 h, before treatment with different concentrations of SOL (0, 50, 100, or 200 μg/ml) for 24 h. Following the addition of 10 μl/well by cell counting kit 8 (CCK8) (#LZ735, Dojindo, Japan), cells were cultured for another 2 h at 37°C. Absorbance was measured at 450 nm by a microplate reader (Cytation3, Bio-Tek, United States). Cell viability was calculated using the following formula:

$$Cell\;viability\;\left(\%\right)=\frac{Number\;of\;treated\;cells}{Number\;of\;control\;cells}\times 100$$

### 2.8 5-Ethynyl-2-deoxyuridine assay

EdU is a thymidine analog that can be incorporated into replicating DNA for the detection of cell proliferation (Salic and Mitchison, 2008). In this study, NSCLC cells were seeded into 96-well plates (4 × 10^3^ cells/well) and incubated for 24 h before treatment with different concentrations of SOL (0, 50, 100, or 200 μg/ml) for 24 h. Cells were then incubated for 2 h with 50 μM EdU (#K1075, APE×BIO, TX, United States) before fixation with 4% formaldehyde (#1810473, Biosharp, Hefei, China) for 30 min. EdU Click buffer was then added for 30 min and cells were stained with Hoechst 33342 for 30 min. EdU-positive cells were imaged with an ImageXpress Micro 4 High-Content Screening System (Molecular Devices, CA, United States) at ×100 magnification. Cell proliferation was analyzed as the percentage of viable cells using the following formula:

$$Cell\;proliferation\;\left(\%\right)=\frac{Number\;of\;EdU\;stained\;cells\;\left(red\right)}{Number\;of\;Hoechst\;33342\;stained\;cells\;\left(blue\right)}\times 100$$

### 2.9 Wound healing assay

A549 and H1299 cells were grown in 6-well plates until 80% confluent. The cell monolayer was then scratched using a sterile pipette tip and the media was removed, before washing with phosphate-buffered saline (PBS, #P1010, Sorlabio, Beijing, China) and replacing with media containing different concentrations of SOL (0, 50, 100, or 200 μg/ml). Cell movements into wound areas were imaged after 0 and 24 h of incubation with a phase-contrast inverted microscope at ×40 magnification.

### 2.10 Transwell invasion assay

A Transwell invasion assay was conducted using a Corning Transwell chamber system (#3442, Corning, NY, United States). Cells were seeded into the upper chamber at a density of 1.5 × 10^5^ cells/chamber in the presence of a Matrigel-precoated membrane (#M8370, Solarbio, Beijing, China) and 200 μl serum-free RPMI 1640. A complete medium (500 μl) containing 10% FBS was added to the bottom chamber. Following incubation for 24 h at 37°C, chambers were washed twice with PBS, fixed with 4% paraformaldehyde, and stained with a 1% crystal violet solution (#C0121, Beyotime, Shanghai, China) at room temperature. Cells were counted in three random fields under a light microscope at ×100 magnification.

### 2.11 Hoechst 33342/PI staining

Cells were seeded into 6-well plates and treated with different concentrations of SOL. Cells were then stained with Hoechst 33342/PI solution (#CA1120, Solarbio, Beijing, China) to identify necrotic cells, following the manufacturer’s instructions. Hoechst 33342 stains cell nuclei, while PI penetrates dying cells due to a loss of cell membrane integrity. Representative images were captured using a fluorescence microscope (Nikon ECLIPSE 80i; Nikon, Tokyo, Japan) at ×40 magnification. The percentage of PI-positive cells was calculated using ImageJ software (National Institutes of Health, Bethesda, MD, United States).

### 2.12 Flow cytometry analysis of apoptosis

A549 and H1299 cells were seeded into 6-well plates (1.5 × 10^5^ cells/well) and treated with different concentrations of SOL for 24 h. For analysis of apoptosis, cells were stained using an Annexin V-FITC/PI Detection Kit (#A211-01, Vazyme, Nanjing, China). A FACS Verse flow cytometer (BD Biosciences, CA, United States) was used for detection. Data acquisition and analysis were performed using FlowJo software (BD Biosciences, CA, United States).

### 2.13 Detection of intracellular ROS levels

NSCLC cells were seeded into 6-well plates (1.5 × 10^5^ cells/well) and treated with SOL for 24 h. The cells harvested after 24 h were centrifuged at 1,500 r/min for 3 min, re-suspended in 500 μl of serum-free medium containing 10 μM DCFH-DA (#S0033, Beyotime, Shanghai, China), incubated in the dark for 20 min under a gentle mixing at 5 min intervals. The cells were subsequently washed with serum-free medium twice, resuspended in 500 μl of PBS, and submitted to the analysis of intracellular oxidative species (ROS) using a FACS Verse flow cytometer under the FITC channel. The quantitation of the test DCF was based on the corresponding mean fluorescence, data analysis was performed using FlowJo software.

### 2.14 Western blot

Cells were seeded into 6-well plates (1.5 × 10^5^ cells/well) and treated with different concentrations of SOL for 24 h. Cells were then lysed on ice for 30 min in radioimmunoprecipitation assay buffer (#9806, Cell Signaling Technology, MA, United States) containing 1:100 protease and phosphatase inhibitor cocktail (#C0001, TargetMol, MA, United States). Lysate protein concentrations were determined using the Bradford protein assay (#500-0205, Bio-Rad, CA, United States), according to the manufacturer’s instructions. Equal amounts of protein (30 μg/sample) were separated by 10% sodium dodecyl sulfate-polyacrylamide gel (#PG11X, Epizyme, Shanghai, China) electrophoresis and transferred onto polyvinylidene difluoride membranes. The membranes were then blocked with 5% non-fat milk for 1 h at room temperature and incubated overnight at 4°C with primary antibodies: anti-mTOR (#2983, Cell Signaling Technology, MA, United States), anti-p-mTOR (#5536, Cell Signaling Technology, MA, United States), anti-PI3K (#4249, Cell Signaling Technology, MA, United States), anti-p-PI3K (#4228, Cell Signaling Technology, MA, United States), anti-AKT (#4691, Cell Signaling Technology, MA, United States), anti-p-AKT (#4060, Cell Signaling Technology, MA, United States), Caspase 3 (#10380-1-AP, Proteintech, Wuhan, China), anti-cleaved-PARP 1 (#56196, SANTA CRUZ, CA, United States) and anti-β-actin (#4970, Cell Signaling Technology, MA, United States) at 1:1,000. After three washes with PBST, membranes were incubated with horseradish peroxidase (HRP)-conjugated secondary antibodies (#7074, #7076, Cell Signaling Technology, MA, United States) for 1 h at room temperature. The membranes were developed using UltraSignal Hypersensitive ECL Chemiluminescent Substrate (#4AW011, 4A Biotech, Beijing, China), and then detected by the ChemiDoc MP Imaging System (Bio-Rad). Bands were quantified using ImageJ software, and expression was normalized to *β*-Actin. Mouse tissue was analyzed following the same protocol after grinding with a tissue grinder (SCIENTZ-48, Ningbo, China).

### 2.15 Animal studies and drug administration

All animal experiments in this study were approved by the Animal Ethics Committee of Southwest Medical University (License No. 20210304-012). Four-week-old male athymic nude mice (Chongqing Tengxin Experimental Animal Co., Ltd., Chongqing, China) were maintained in pathogen-free conditions. A549 cells (1 × 10^6^/mouse in 0.1 ml PBS) were injected subcutaneously into the right flank; after 12 days, tumor volumes were between 75 and 100 mm^3^. Mice were randomly divided into five groups (6 mice/group): a control group (0.9% NaCl with 15% ethanol), SOL-treated groups (25, 50, and 100 mg/kg), and a paclitaxel (PTX) group (10 mg/kg, positive control group). Mice were sacrificed after intraperitoneal (I.P.) administration for 12 consecutive days, and tumor tissues were collected for the detection of PI3K/AKT/mTOR activation by Western blot and immunohistochemistry.

### 2.16 Tumor volumes and weights

Tumor volumes were measured daily with a Vernier caliper. Tumor volumes were determined using the following formula (Hou et al., 2020):

$$Volume=\frac{{width}^{2}\times length}{2}$$

After 12 days of the administration, mice were sacrificed, autopsies were performed, and tumor xenografts were isolated, analyzed, and weighed. The tumor inhibition rate (IR) was calculated as follows:

$$IR=\frac{1-mean\left({tumor\;weight}_{drug}\right)}{mean\left({tumor\;weight}_{control}\right)}\times 100$$

### 2.17 Peripheral blood and platelet assay

After 12 days, 40 μl whole blood samples were collected from the retro-orbital venous plexus and diluted with 160 μl diluent (#8169, Sysmex, Kobe, Japan) for blood cell analysis. Complete blood cell counts were determined using an automatic hematology analyzer (XT-2000iV, Sysmex, Kobe, Japan).

### 2.18 Hematoxylin and eosin staining

Tumors and organs including the heart, liver, spleen, lung, and kidney were fixed with 4% paraformaldehyde, embedded in paraffin, and sectioned for H&E staining (#G1003, Servicebio, Wuhan, China). Stained tumor and organ slices were imaged using an upright optical microscope (Nikon Eclipse E100, Nikon, Tokyo, Japan) at ×200 magnification.

### 2.19 Immunohistochemistry

Cell proliferation in tumors was assessed by immunohistochemical analysis of Ki67 expression. Briefly, paraffin sections were dewaxed in xylene, washed twice, rehydrated with 75%–85% ethanol, and washed three times with distilled water. After adding the citric acid repair solution (#G1202, Servicebio, Wuhan, China), the slices were placed in a microwave oven and fixed for 15 min. Slices were then cooled and washed three times with 5 min PBS washes, before 3% H_2_O_2_ was added and slices were again washed three times with PBS for 25 min. Sections were then blocked with 5% blocking serum for 30 min before incubating with Ki67 primary antibody (#ab15580, Abcam, Cambridge, UK), at 4°C overnight. The following day, anti-rabbit/mouse HRP-labeled polymer was added for 1 h at 37°C. Then, the pre-formed developer diaminobenzidine working solution was added, incubated for 5 min, and rinsed with distilled water. Slices were then hematoxylin-counterstained for 3 min, and rinsed with distilled water. Finally, the slices were dehydrated, sealed, and mounted with neutral gum. Representative images were captured using an upright optical microscope.

### 2.20 Statistical analysis

The data acquired in this study were statistically analyzed using GraphPad Prism 8.0 (GraphPad Software Inc., CA, United States). The data are expressed as the means ± standard deviation $\left(\bar{x}\pm SD\right)$ . Statistical significance was assessed using one-way analysis of variance (ANOVA). In all cases, a *p*-value < 0.05 was considered to indicate a significant difference.

## 3 Results

### 3.1 Network pharmacology analysis revealed the potential mechanism of *Sanguisorba officinalis* L*.* in NSCLC

**FIGURE 1:**
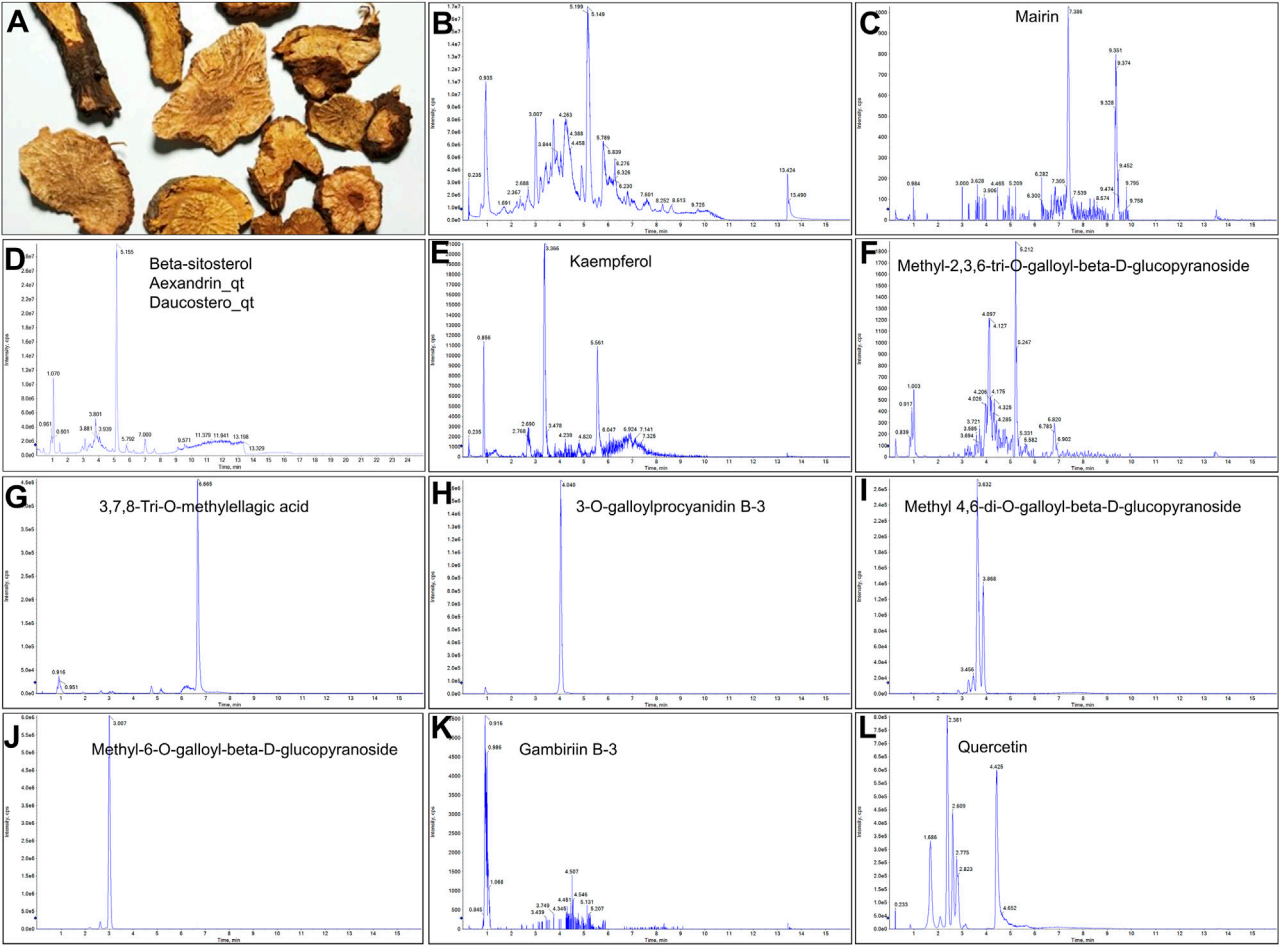
UPLC-MS-QTOF chromatograms of the 12 compounds detected in samples. **(A)** Dry rhizomes of *Sanguisorba officinalis* L.; **(B)** Total ion flow diagram of 70% ethanol extract of *Sanguisorba officinalis* L.; **(C)** Mairin; **(D)** Beta-sitosterol or Alexandrin_qt or Daucostero_qt; **(E)** Kaempferol; **(F)** Methyl-2,3,6-tri-O-galloyl-beta-D-glucopyranoside; **(G)** 3,7,8-Tri-O-methylellagic acid; **(H)** 3-O-galloylprocyanidin B-3; **(I)** Methyl 4,6-di-O-galloyl-beta-D-glucopyranoside; **(J)** Methyl-6-O-galloyl-beta-D-glucopyranoside; **(K)** Gambiriin B-3; **(L)** Quercetin.

**FIGURE 2:**
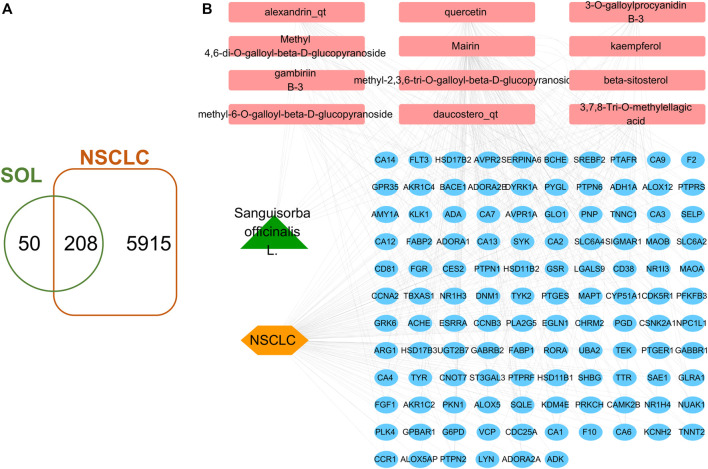
Venn diagram and disease-drug-target network of SOL and NSCLC. **(A)** Venn diagram of SOL targets and NSCLC targets. **(B)** SOL-compounds-NSCLC-targets network. Among them, the green node represents SOL, the pink nodes represent the 12 compounds in SOL, the orange node represents NSCLC, and the blue nodes represent their common targets.

**FIGURE 3:**
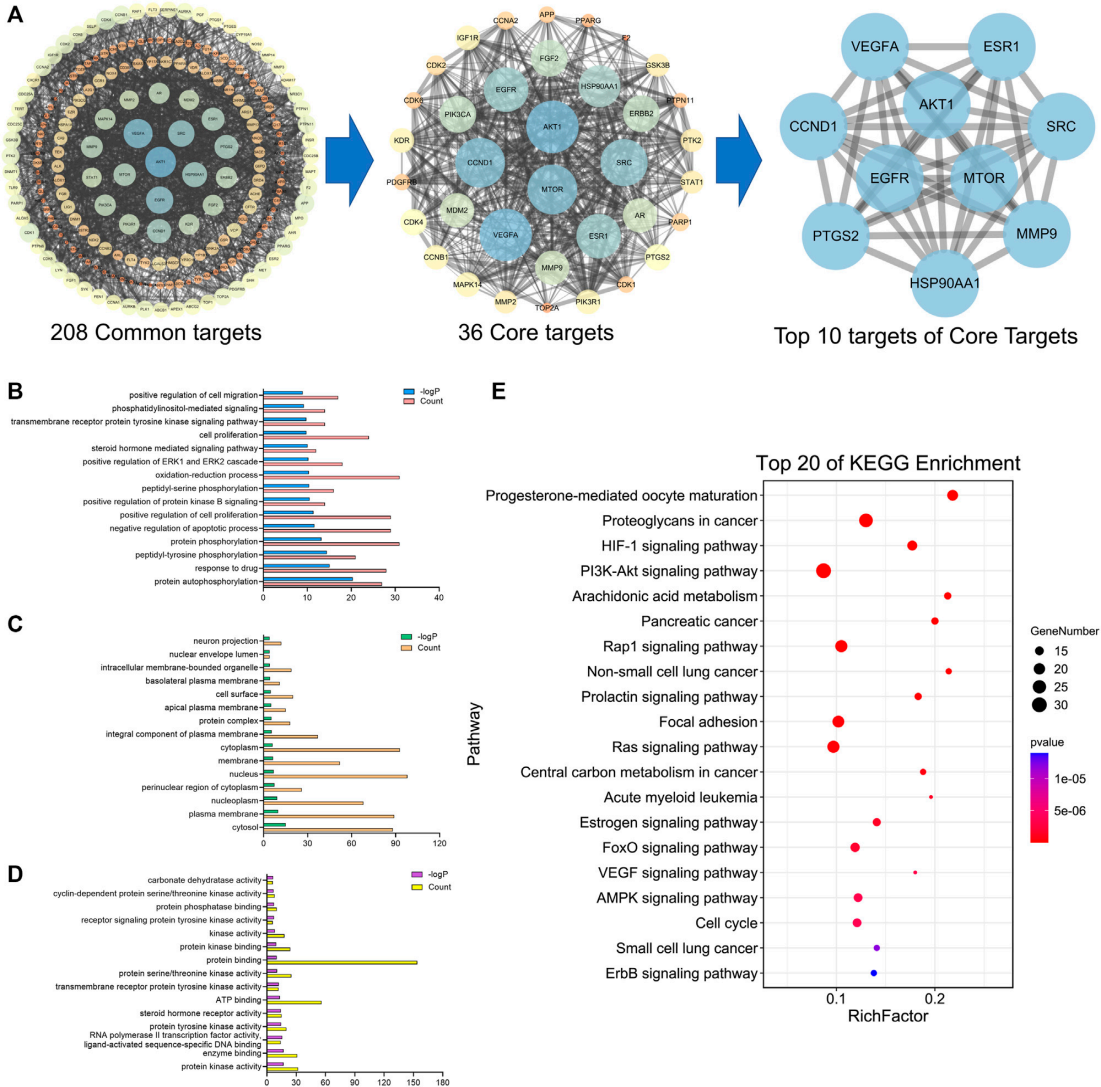
PPI and enrichment analyses based on network pharmacology. **(A)** Protein-protein interaction (PPI) network of bioactive compounds of SOL against NSCLC. GO enrichment analysis of common targets for SOL against NSCLC. **(B)** Biological process, **(C)** cellular components, and **(D)** molecular function were shown in histograms. **(E)** Top 20 enriched KEGG pathways of the common targets for SOL against NSCLC. The color scales indicated the different thresholds of adjusted *p*-values, and the sizes of the dots represented the gene count of each term.

A total of 41 reported active compounds of SOL were retrieved by searching the databases, and 12 bioactive compounds were acquired by OB and DL (Table 1) and verified *via* UPLC-MS (Figure 1). 258 putative targets of the 12 compounds in SOL and 6,123 targets of NSCLC were obtained. Through analysis of intersections, 208 common targets were identified as potential targets of SOL in NSCLC (Figure 2A). A drug-disease-target network was constructed based on the information on targets (Figure 2B). A protein-protein interaction (PPI) network of the 208 common targets was established using the STRING database and Cytoscape software (Figure 3A). According to the screening threshold criteria of degree, betweenness centrality, and closeness centrality, 36 core targets were acquired (Figure 3A). The top 10 targets of these 36 core targets were AKT1, VEGF, EGFR, SRC, HSP90AA1, CCND1, PTGS2, ESR1, mTOR, and MMP9 (Figure 3A; Table 2). To elucidate the underlying mechanisms of SOL activity in NSCLC, GO and KEGG pathway enrichment analyses were performed using DAVID. GO analysis demonstrated that the mechanism were mainly related to protein autophosphorylation, protein phosphorylation, and cell proliferation (biological processes); protein kinase activity, enzyme binding, and protein phosphatase binding (molecular function); and the cytoplasm, membrane, and nucleus (cellular components) (Figures 3B–D). The KEGG pathway enrichment analysis showed that the core targets were significantly enriched in cancer-related pathways such as the PI3K-AKT, HIF-1, FoxO, VEGF, and AMP-activated protein kinase signaling pathways (Figure 3E). Combined with the ranking of core targets, the PI3K-AKT signaling pathway was the most related pathway of SOL in NSCLC.

**TABLE 1:** Active compounds of *Sanguisorba officinalis* L.

Mol ID	Compounds	OB (%)	DL	MW	Structure
MOL000211	Mairin	55.38	0.78	456.78	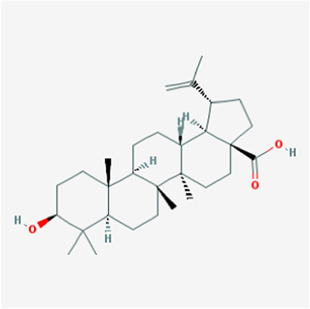
MOL000358	beta-sitosterol	36.91	0.75	414.79	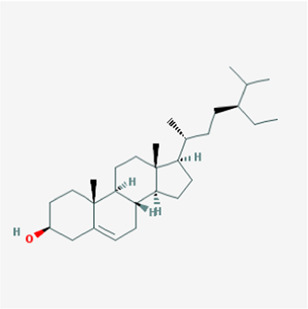
MOL000422	kaempferol	41.88	0.24	286.25	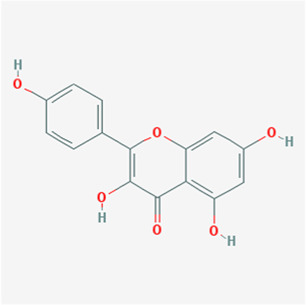
MOL005399	alexandrin_qt	36.91	0.75	414.79	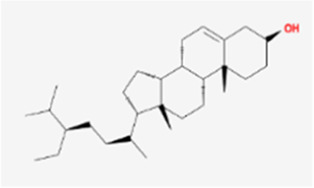
MOL005853	methyl-2,3,6-tri-O-galloyl-beta-D-glucopyranoside	44.95	0.67	654.68	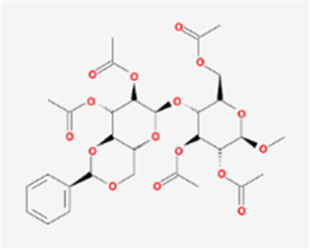
MOL005858	3,7,8-Tri-O-methylellagic acid	37.54	0.57	344.29	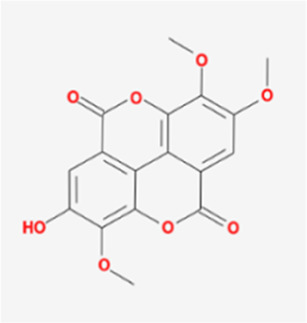
MOL005860	3-O-galloylprocyanidin B-3	30.06	0.33	730.67	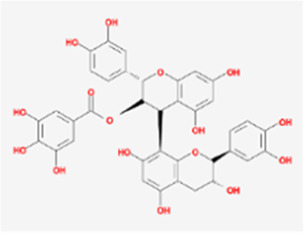
MOL005862	Methyl 4,6-di-O-galloyl-beta-D-glucopyranoside	48.07	0.68	498.43	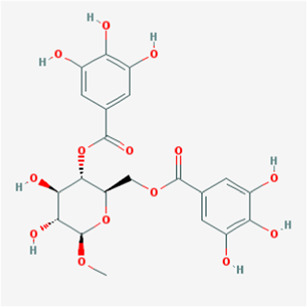
MOL005864	methyl-6-O-galloyl-beta-D-glucopyranoside	44.85	0.29	346.32	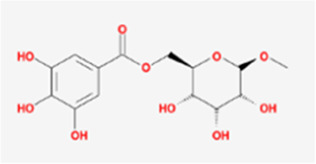
MOL005869	daucostero_qt	36.91	0.75	414.79	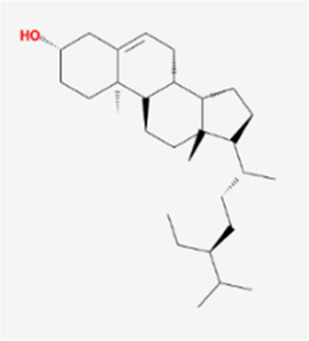
MOL005883	gambiriin B-3	34.99	0.75	546.56	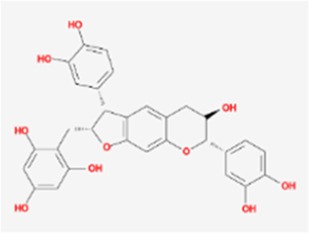
MOL000098	quercetin	46.43	0.28	302.25	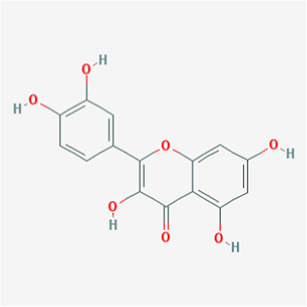

**TABLE 2:** Core targets of *Sanguisorba officinalis* L. against NSCLC.

Name	Betweenness centrality	Closeness centrality	Degree
AKT1	0.113917	0.669967	106
VEGFA	0.09856966	0.64240506	97
EGFR	0.06181598	0.62269939	87
SRC	0.06079439	0.61702128	82
HSP90AA1	0.05083103	0.59356725	74
CCND1	0.03171206	0.57670455	70
PTGS2	0.04973657	0.58333333	68
ESR1	0.03225588	0.57344633	63
MTOR	0.01563979	0.56232687	60
MMP9	0.02324461	0.5501355	57

### 3.2 *Sanguisorba officinalis* L*.* exhibited promising anti-NSCLC activity

**FIGURE 4:**
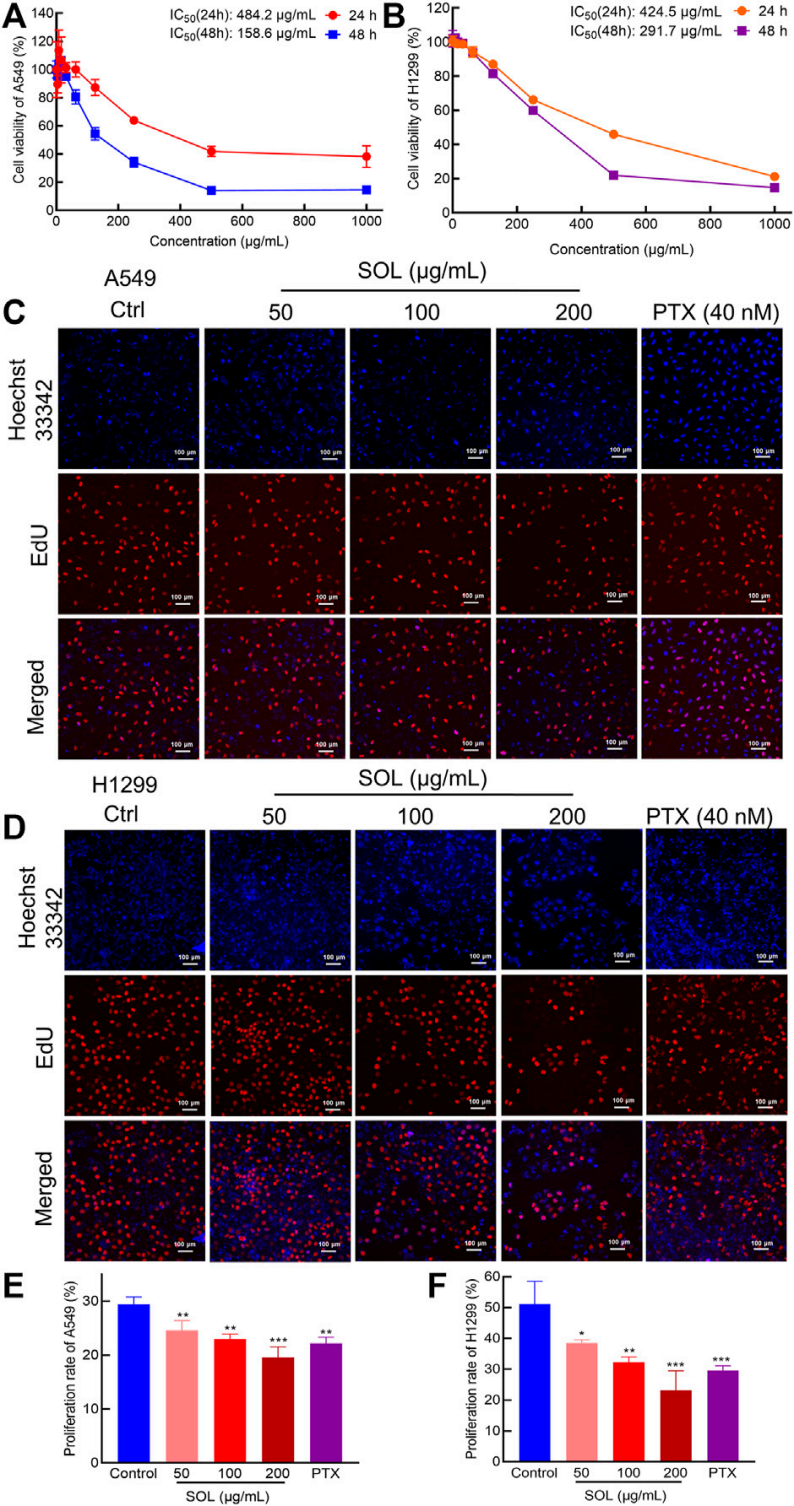
Proliferation inhibition of SOL in NSCLC cells. CCK8 assay was conducted in A549 **(A)** and H1299 **(B)** cells treated with SOL for 24 h and 48 h. Cell viability curves and IC_50_ values were shown, respectively. EdU assay was conducted in A549 **(C)** and H1299 **(D)** cells treated with SOL for 24 h. Blue represents the cell nucleus, and red represents DNA replication. SOL significantly decreased the proportion of EdU-positive cells compared with that of control A549 **(E)** and H1299 **(F)** cells. Magnification: ×100, scale bar in white: 100 µm. Bars in the histograms represent the mean ± SD from three independent experiments. Compared with Ctrl group, *p < 0.05, **p < 0.01, and ***p < 0.001.

Analysis by CCK8 and EdU assays demonstrated that SOL inhibited the growth and proliferation of A549 and H1299 cells in a dose-dependent manner. CCK8 analysis revealed that SOL significantly inhibited the viability of A549 and H1299 cells (Figures 4A,B), with IC_50_ 484.2 and 424.5 μg/ml at 24 h, 158.6 and 291.7 μg/ml at 48 h for A549 and H1299 cells, respectively. In the EdU assay, SOL showed remarkable cytotoxicity in A549 and H1299 cells (Figures 4C,D). Calculating the ratio of EdU-positive cells to total cells, we found that the percentage of SOL-induced cell death in A549 and H1299 cells (Figures 4E,F) increased in a dose-dependent manner. Immunohistochemical analysis of Ki67 expression in tumor tissues also revealed that tumor cell proliferation was inhibited by SOL (Figure 12E). Furthermore, the downregulation of cyclin D1 expression in tumor tissues revealed that the cell cycle was obstructed to inhibit cell proliferation by SOL (Supplementary Figure S1).

**FIGURE 5:**
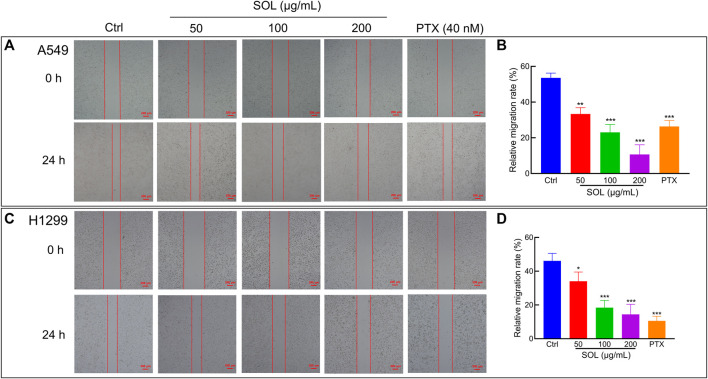
Cell migration of SOL in NSCLC cells. SOL decreased the migration of A549 **(A)** and H1299 **(C)** cell lines, as shown by wound healing assays. The statistical results of the relative migration rate of A549 **(B)** and H1299 **(D)** cells were shown in this figure, respectively. Magnification: ×100, scale bar in red: 100 µm. Bars in the histograms represent the mean ± SD from three independent experiments. Compared with Ctrl group, *p < 0.05, **p < 0.01, and ***p < 0.001.

**FIGURE 6:**
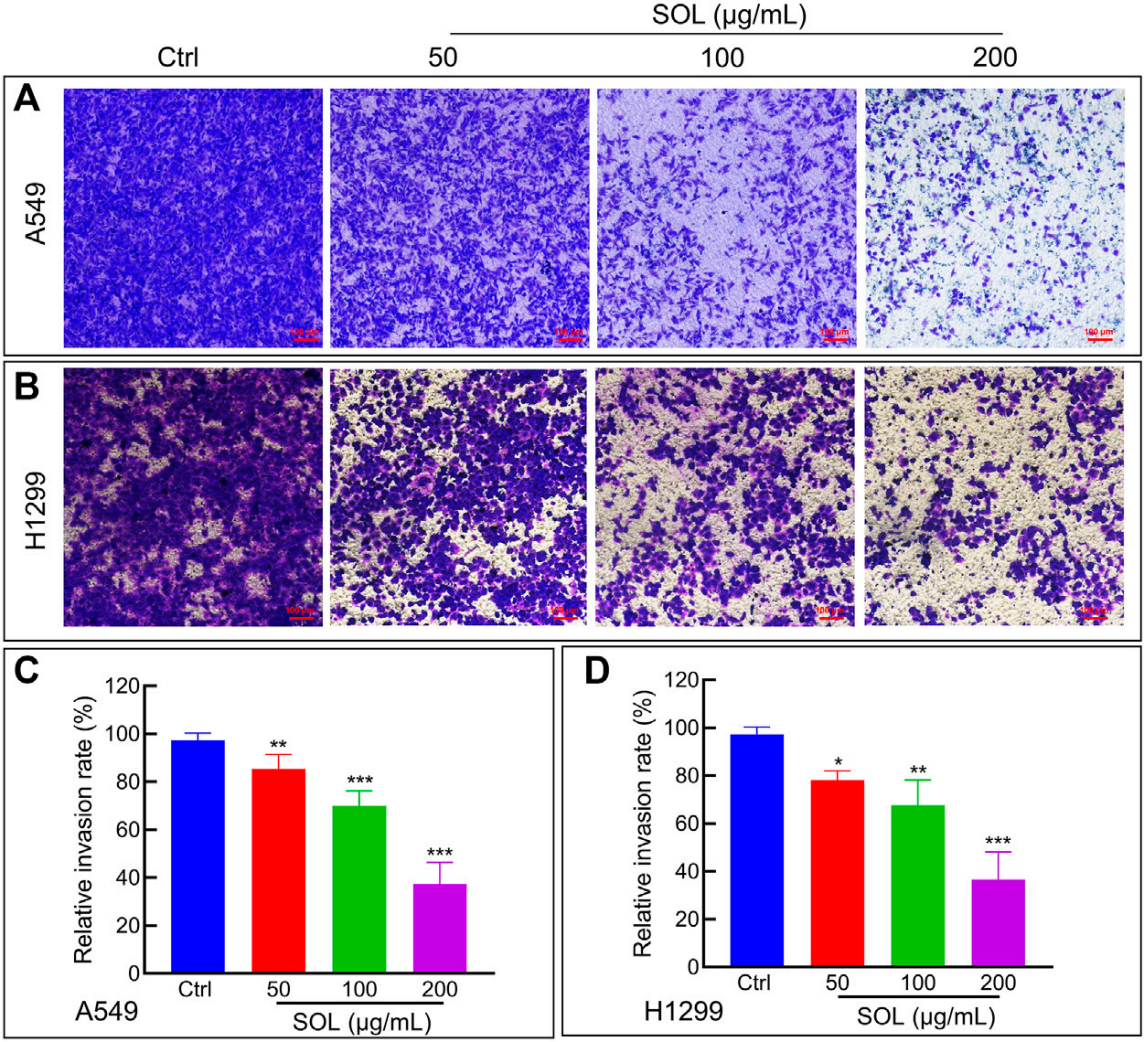
Cell invasion of SOL in NSCLC cells. SOL decreased the invasion of A549 **(A)** and H1299 **(B)** cell lines, as shown by transwell assays, respectively. Magnification: ×100, Scale bar in red: 100 µm. The statistical results of the relative invasion rate of A549 **(C)** and H1299 **(D)** cells were shown in this figure, respectively. Bars in the histograms represent the mean ± SD from three independent experiments. Compared with Ctrl group, *p < 0.05, **p < 0.01, and ***p < 0.001.

To explore the effect of SOL on the migration and invasion of A549 and H1299 cells, wound healing and Transwell assays were performed. Compared to the control group, the migration of A549 and H1299 cells was markedly inhibited in the SOL treatment groups (Figure 5). In the Transwell assay, the invasion of SOL-treated A549 and H1299 cells (Figure 6) was inhibited in a dose-dependent manner. Immunohistochemical analysis of MMP9 expression in tumor tissues revealed that invasion of the tumor was inhibited by SOL (Supplementary Figure S2).

### 3.3 *Sanguisorba officinalis* L*.* accelerated apoptosis in NSCLC cells

**FIGURE 7:**
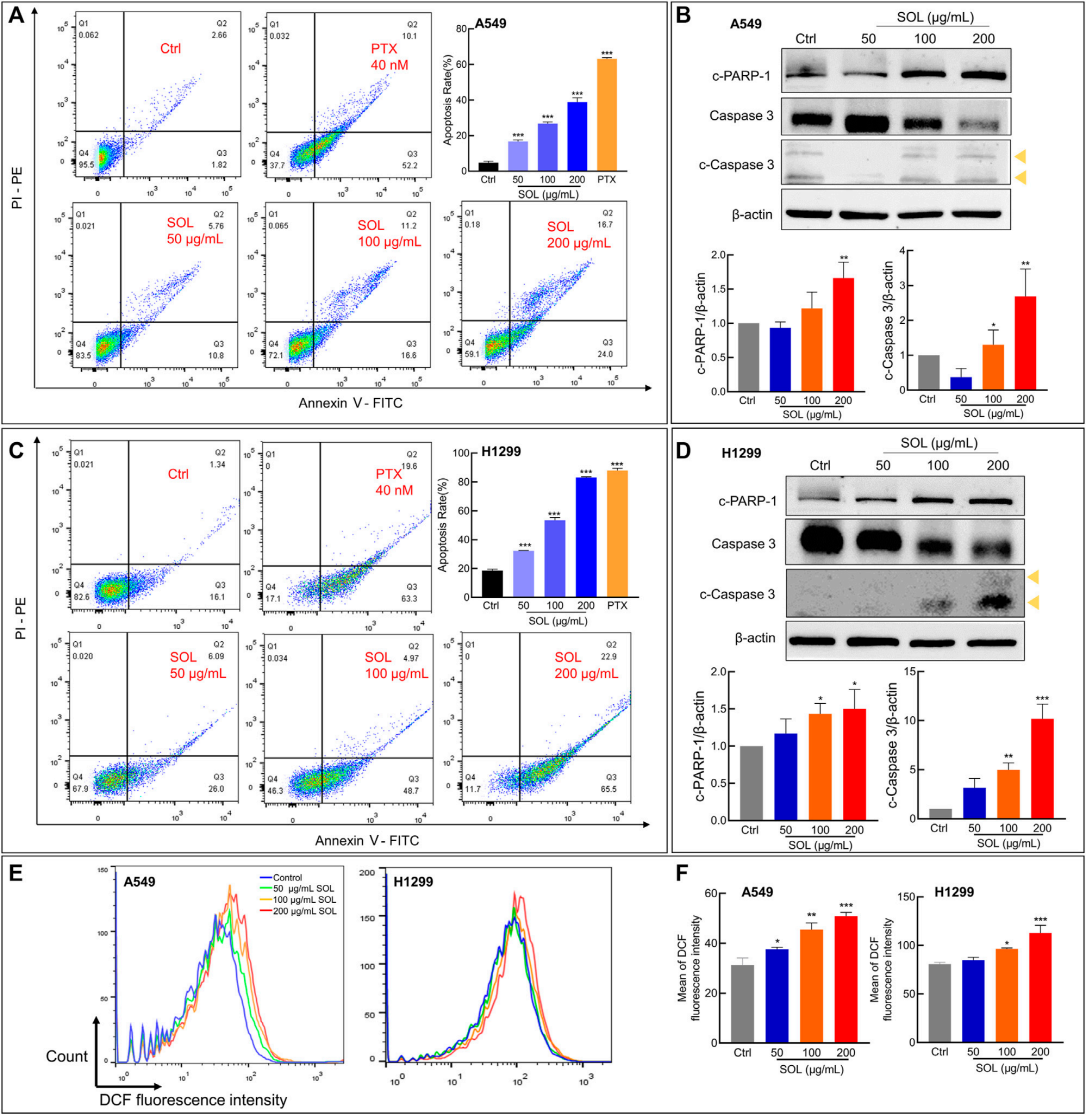
Cell apoptosis and ROS level of SOL in NSCLC cells. SOL increased the apoptosis of A549 **(A)** and H1299 **(C)** cell lines, as shown by FACS assays. The statistical results of the apoptosis rate of A549 and H1299 cells were shown, respectively. The apoptosis-related protein levels of c-PARP-1, caspase 3, and c-caspase 3 in A549 cells **(B)** and H1299 cells **(D)** were detected by western blotting. The yellow arrows indicated the location of the two cleaved bands of Caspase-3. Bars in the histograms represent the mean ± SD from three independent experiments. Compared with Ctrl group, *p < 0.05, **p < 0.01, and ***p < 0.001. **(E)** Intracellular ROS level in NSCLC cells using flow cytometry analysis. **(F)** Quantitative analysis of ROS level in NSCLC cells. Bars in the histograms represent the mean ± SD from three independent experiments. Compared with Ctrl group, *p < 0.05, **p < 0.01, and ***p < 0.001.

**FIGURE 8:**
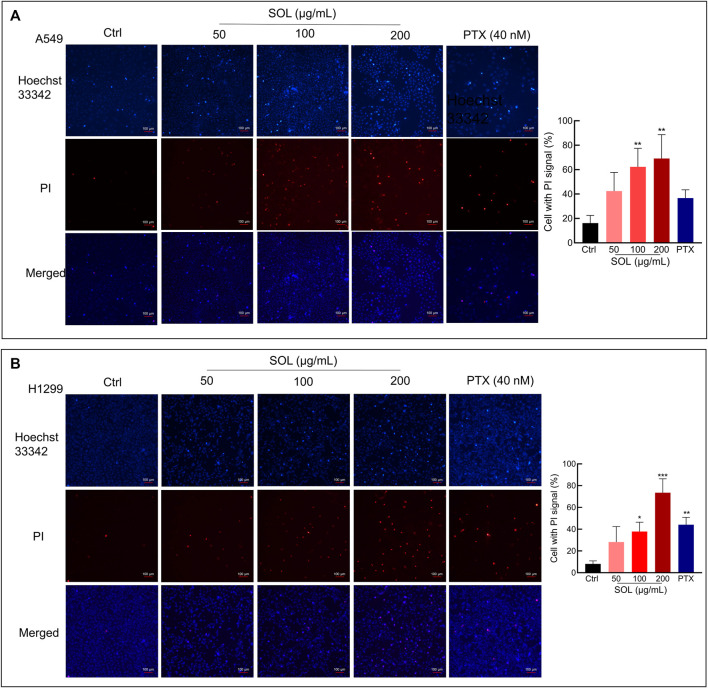
Hoechst 33342/PI staining of SOL on NSCLC cells. A549 cells **(A)** and H1299 cells **(B)** were treated with SOL at the indicated concentration for 24 h. The cells were then stained with PI (red, staining dying cells) plus Hoechst 33342 (blue, staining all cells), and observed by fluorescent microscopy. The bar chart indicates the percentage of cells with a PI signal compared to the blue signal. Magnification: ×100, scale bar in white: 100 µm. Bars in the histograms represent the mean ± SD from three independent experiments. Compared with Ctrl group, *p < 0.05, **p < 0.01, and ***p < 0.001.

SOL treatment generated a significant increase in the Annexin V-positive population as determined by flow cytometry (Figures 7A,C). Cleavage of caspase-3 and poly (ADP-ribose) polymerase-1 (PARP-1), considered apoptosis markers, was induced by SOL treatment (Figures 7B,D; Supplementary Figure S3), so as in tumors of A549-bearing athymic nude mice (Figures 12B,D). To reveal the type of cell death induced by SOL, A549 and H1299 cells, they were doubly stained with Hoechst 33342/PI. Hoechst 33342 staining revealed that SOL treatment decreased the density of A549 and H1299 cells (Figures 8A,B), while PI staining revealed that the proportion of cells undergoing SOL-induced lytic cell death (PI-positive cells) increased in a dose-dependent manner.

ROS generation plays a pivotal role in regulating the process of cancers (Srinivas et al., 2019). ROS induces apoptosis if produced excessive amounts (Sies and Jones, 2020). The results from the flow cytometry assay using the DCFH-DA probe showed that SOL induced excessive ROS production in NSCLC cells, which was in a concentration-dependent manner (Figures 7E,F). This result consistently confirmed oxidative stress was induced by SOL in NSCLC cells, thus resulting in the acceleration of apoptosis.

### 3.4 *Sanguisorba officinalis* L*.* suppressed tumor growth in A549-bearing athymic nude mice

**FIGURE 9:**
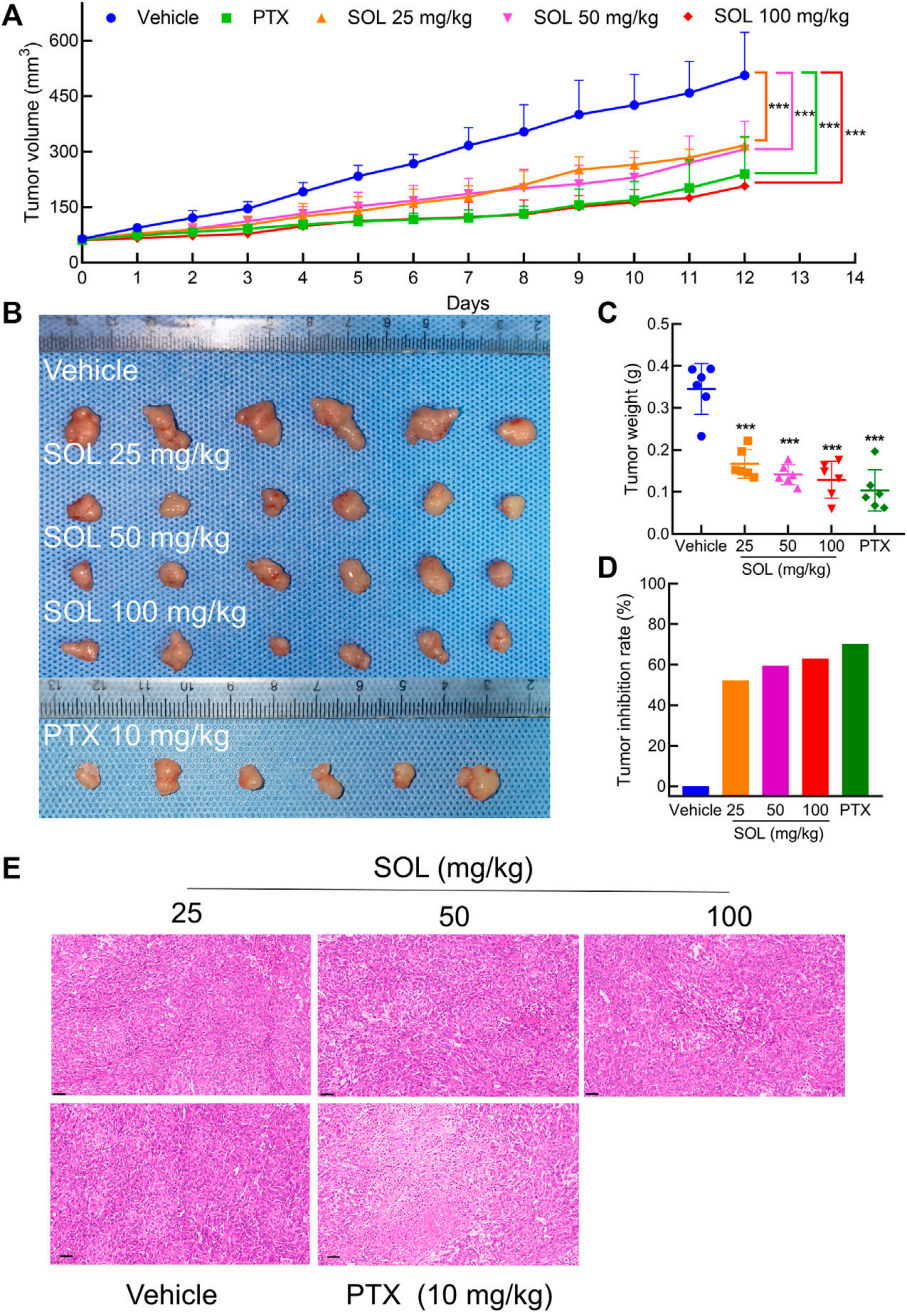
SOL inhibited tumor growth *in vivo*. **(A)** Statistical results of tumor volume change in mice during 12 days of continuous administration. Bars in the line chart represent the mean ± SD in each group of the 12th day. Compared with Vehicle group, ***p < 0.001. **(B)** Tumor volume in mice after 12 days of continuous administration. **(C)** Statistical results of tumor weight in mice after 12 days of continuous administration. Bars in the chart represent the mean ± SD in each group. Compared with Vehicle group, ***p < 0.001. **(D)** Statistical results of tumor inhibition rate in mice after 12 days of continuous administration. **(E)** H&E staining images of tumor tissues were dissected from each group on the 12th day. Magnification: ×200, scale bar in black: 50 μm.

Based on the potent anti-proliferative effect of SOL *in vitro*, we further validated its inhibitory effect on tumor growth in A549-bearing athymic nude mice. After tumor establishment on the 12th day, mice were I.P.-administered PTX (10 mg/kg) or SOL (25, 50, or 100 mg/kg) daily from days 13–24. After drug administration, tumor volumes were found to be significantly reduced in the SOL groups compared to the vehicle group (Figures 9A,B). Tumor weights were remarkably decreased (Figure 9C), and the rate of tumor inhibition also increased significantly in the SOL groups (Figure 9D). In addition, H&E staining of tumor tissues showed that the proliferation of tumor cells was inhibited (Figure 9E).

### 3.5 Normal cells and tissues were less sensitive to *Sanguisorba officinalis* L*.*

**FIGURE 10:**
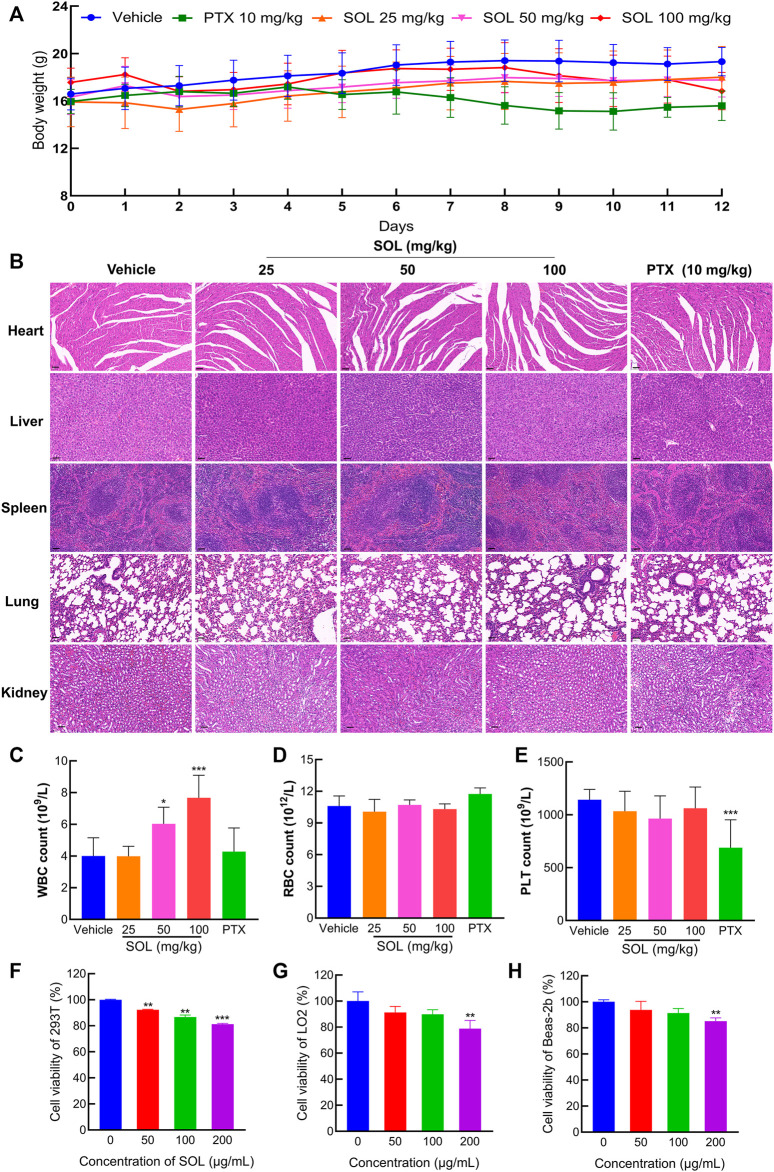
The safety of SOL in normal cells and tissues. **(A)** The body weight of mice changed during 12 days of continuous administration. **(B)** H&E staining images of major organs (heart, liver, spleen, lung, and kidney) were dissected from each group on the 12th day after photothermal treatment. Magnification: ×200, scale bar in black: 50 µm. Level of white blood cells **(C)**, red blood cells **(D)**, and platelets **(E)** after 12 days of administration. Bars in the histograms represent the mean ± SD in each group. Compared with Vehicle group, ***p < 0.001. CCK8 assay was conducted in normal cells 293T **(F)**, LO2 **(G)**, and Beas-2b **(H)** cells treated with SOL for 24 h. Bars in the histograms represent the mean ± SD from three independent experiments. Compared with Ctrl group, **p < 0.01 and ***p < 0.001.

To test the safety of SOL in normal cells, 293T, LO2, and Beas-2b cell lines were employed *in vitro*. The results showed that the concentrations of SOL used in the study induced no significant toxicity on the liver, kidney, and lung cells (Figures 10F–H). Besides, during the experiments in A549-bearing athymic nude mice, no significant change in body weight was found (Figure 10A). To further confirm the safety of SOL, mice were dissected on the 12th day post-treatment and their major organs were subjected to histological analysis. Compared with the control group, no significant organ damage or inflammation was found, further confirming the safety and low toxicity of SOL (Figure 10B; Supplementary Figure S4). Peripheral blood tests showed that no significant changes were observed in red blood cells and platelet numbers (Figures 10D,E). Interestingly, the white blood cell count in the SOL group increased significantly in a dose-dependent manner (Figure 10C).

### 3.6 *Sanguisorba officinalis* L*.* suppressed NSCLC *via* downregulating the PI3K/AKT/mTOR pathway

**FIGURE 11:**
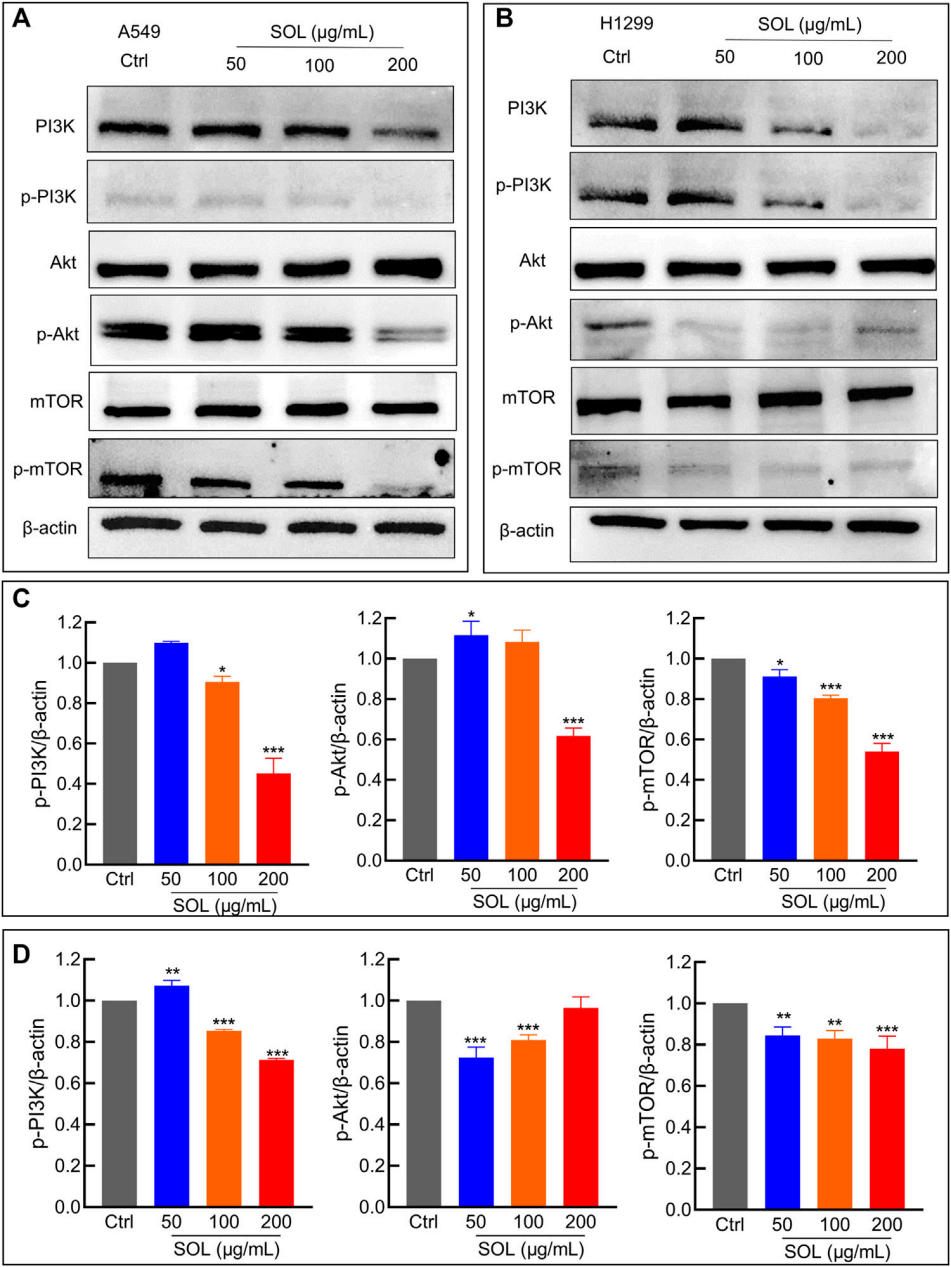
SOL suppressed NSCLC *via* the PI3K/AKT/mTOR signaling pathway. **(A)** A549 and **(B)** H1299 cells were treated with SOL (0, 50, 100, 200 μg/ml) for 24 h. The cell protein was then harvested for detecting PI3K, AKT, mTOR, and *β*-Actin by Western blotting. **(C,D)** Histograms indicated the relative density of these proteins to *β*-Actin. Bars in the histograms represent the mean ± SD from three independent experiments. Compared with Ctrl group, *p < 0.05, **p < 0.01 and ***p < 0.001. Compared with Ctrl group, *p < 0.05, **p < 0.01, and ***p < 0.001.

**FIGURE 12:**
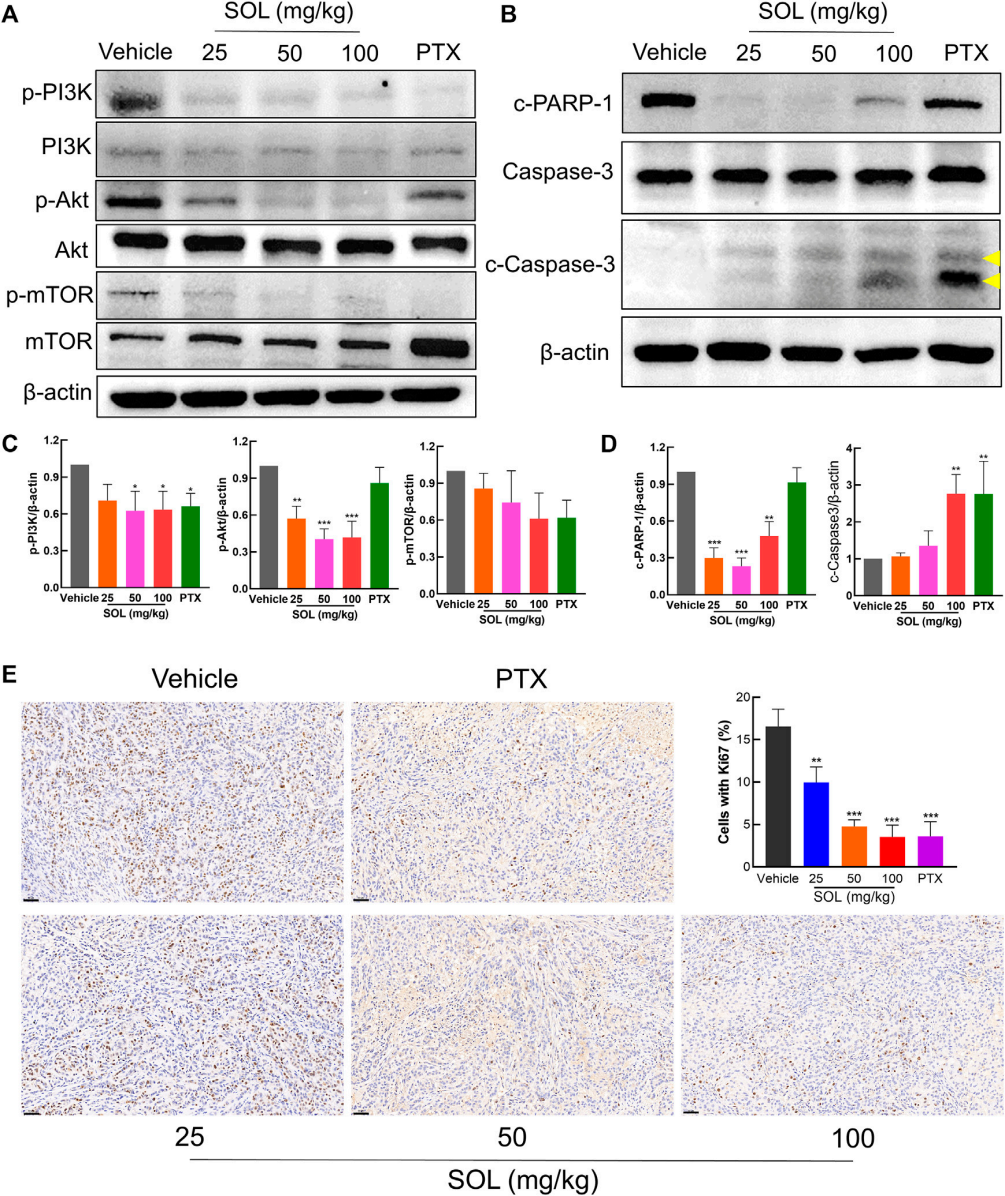
SOL exhibited the anti-NSCLC activity in A549-bearing athymic nude mice. **(A)** Tumor tissue lysates were analyzed by Western blot for mTOR, PI3K, AKT, and *β*-Actin. **(B)** Tumor tissue lysates were analyzed by Western blot for cleaved PARP-1 (c-PARP-1), Caspase-3, cleaved Caspase-3 (c-Caspase-3), and *β*-Actin. The yellow arrows indicated the location of the two cleaved bands of Caspase-3. **(C)** Histograms indicated the relative density of the proteins (p-mTOR, p-PI3K, and p-AKT) to *β*-Actin. Bars in the histograms represent the mean ± SD in each group. Compared with Vehicle group, *p < 0.05, **p < 0.01, and ***p < 0.001. **(D)** Histograms indicated the relative density of the proteins (c-PARP-1 and c-Caspase-3) to *β*-Actin. Bars in the histograms represent the mean ± SD in each group. Compared with Vehicle group, **p < 0.01 and ***p < 0.001. **(E)** The expression of Ki67 in the tumor tissue of A549-bearing athymic nude mice was analyzed by the immunohistochemistry method. Magnification: ×200, scale bar in black: 50 µm. Bars in the histogram represent the mean ± SD in each group. Compared with Vehicle group, **p < 0.01 and ***p < 0.001.

Network pharmacology analysis suggested that AKT was the top-ranked target and PI3K-AKT was the most significant signaling pathway of SOL activity in NSCLC. Therefore, we further assessed the expression levels of PI3K, AKT, mTOR, and their phosphorylated counterparts by Western blot. Treatment of A549 and H1299 cells with SOL showed the apparent repression of PI3K, AKT and mTOR phosphorylation in a dose-dependent manner (Figure 11). Likewise, the protein expressions of p-PI3K, p-AKT, and p-mTOR were found to be significantly downregulated in the tumors of the mouse xenograft model (Figures 12A,C).

## 4 Discussion

NSCLC is the most common lung cancer, most of which are diagnosed at a late stage and the five-year survival rate is low (Sung et al., 2021). However, drug resistance and drug toxicity are the main problems during the treatment of NSCLC. Searching for effective and safe therapeutic drugs to treat NSCLC becomes a hot topic nowadays. Various extracts from TCMs have shown anti-NSCLC effects in recent years (Zhou et al., 2017; Wang et al., 2018; Zhang X et al., 2020), and modern studies have shown that extracts of SOL have antitumor effects (Choi et al., 2012; Liu et al., 2016; Wang N et al., 2020; Jiang et al., 2021). These findings suggested that SOL has potential against NSCLC. Thus, we explored the relationship between SOL and NSCLC to verify the hypothesis.

Network pharmacology is the comprehensive exploration of compounds, pathways, genes, and proteins (Tang and Aittokallio, 2014; Boezio et al., 2017). As an auxiliary means of experimental research, network pharmacology provides a reference for predicting the relationship between TCMs and diseases (Luo et al., 2020). Therefore, network pharmacology was employed to reveal the possibility and mechanisms underlying SOL activity in NSCLC. In the present study, databases and software were used to acquire the common targets of SOL and NSCLC. These targets are associated with cell proliferation, cell migration, apoptosis, and protein phosphorylation, which are closely related to tumorigenesis. Among the potential targets, AKT is top-ranked. AKT (Protein kinase B, PKB) is a serine/threonine kinase that plays a key in regulating cell survival and tumor formation. Activation of AKT promotes cell proliferation and suppresses apoptosis (Manning and Toker, 2017). In addition to identifying the targets of SOL in NSCLC, we obtained 10 potential pathways using KEGG pathway enrichment analysis. Of these pathways, the PI3K/Akt signaling pathway caught our attention not only because of its top rank among the enriched pathways but also because of its linkage to AKT as a major mediator. The PI3K/AKT/mTOR pathway plays an important role in cell growth and proliferation. Phosphatidylinositol 3-kinase (PI3K), phosphorylating the inositol ring of phosphatidylinositol, plays a vital role in a variety of human cancers including NSCLC (Fruman et al., 2017). AKT is the major downstream target of PI3K. The mechanistic target of rapamycin (mTOR) is one of the downstream of AKT, which is related to cell proliferation, growth, and apoptosis (Saxton and Sabatini, 2017). Moreover, the constitutive activation of the PI3K/AKT/mTOR signaling pathway occurs in 90% of NSCLC cell lines (Zhao et al., 2015). ROS is the specific event upstream of the inhibition of the PI3K/AKT signaling pathway and induction of apoptosis (Liu et al., 2021). ROS can induce DNA damage and increase the mutation rate, contributing to tumorigenesis (Harris and DeNicola, 2020). Importantly, ROS leads to caspase-3 activation (Onodera et al., 2018). Consistently, we have found an elevation in the ROS level in SOL-induced NSCLC cells. More importantly, PARP-1, one of the core targets identified from network pharmacology analysis, plays a key role in cell apoptosis (Yu et al., 2002; Schiewer et al., 2018). And caspase-3 is the execution of apoptosis (Eto et al., 2012). In this study, the cleavage of PARP-1 and caspase-3 were dramatically enhanced *in vitro* and *in vivo*. Therefore, SOL resulted in caspase- and ROS-dependent apoptosis through downregulating the PI3K/AKT/mTOR signaling pathway in NSCLC.

**FIGURE 13:**
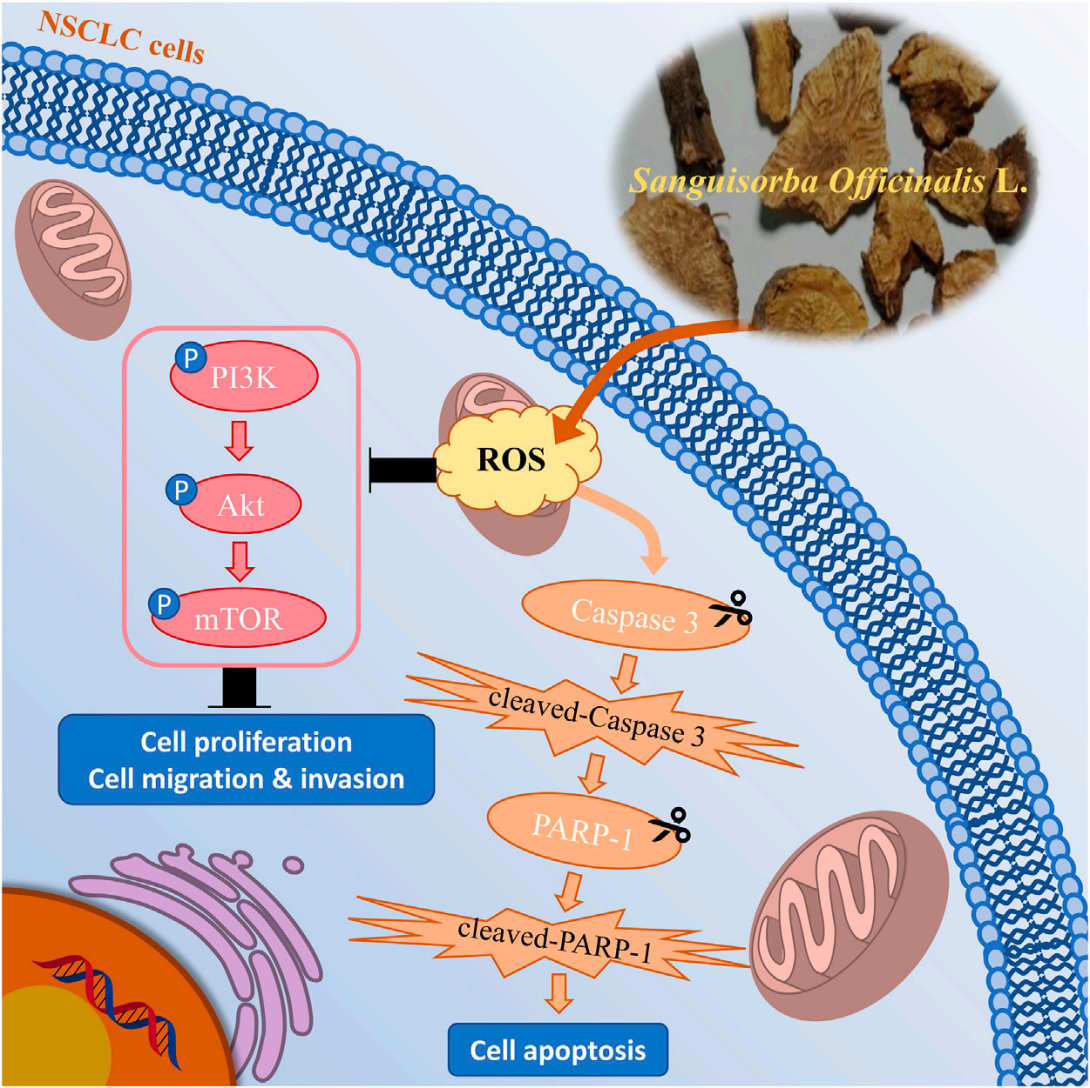
Mechanism chart of the study. SOL downregulated the PI3K/AKT/mTOR signaling pathway to suppress NSCLC cell growth, including cell proliferation, migration, and invasion, by inducing the production of ROS. Simultaneously, SOL induced ROS production, thus activated caspase-3 and PARP-1 to promote cell apoptosis. A “P” in the circle represents phosphorylation. Scissors indicate the cleaved process of caspase 3 and PARP-1.

Collectively, we demonstrated that SOL downregulated the PI3K/AKT/mTOR signaling pathway to suppress NSCLC cell growth (i.e.,: cell proliferation, migration, and invasion) and activated caspase-3 and PARP-1 to promote cell apoptosis by inducing ROS production (Figure 13). To our knowledge, this is the first study showing that SOL suppressed NSCLC both *in vitro* and *in vivo* based on network pharmacology. This research provides a positive preclinical study for the development of potential antitumor TCM drugs. However, the limitation of the present study is the unknown specific active components of SOL in NSCLC, which we will explore in the following research. Mechanistically, predictions of other pathways require further validation to improve this study.

## 5 Conclusion

In summary, the present study predicted the mechanism of SOL in NSCLC and determined conclusively that SOL downregulated the PI3K/AKT/mTOR signaling pathway to suppress NSCLC.

## Data Availability

The original contributions presented in the study are included in the article/Supplementary Material, further inquiries can be directed to the corresponding authors.
