# Supplementary material for: Sanguisorba officinalis L. suppresses non-small cell lung cancer via downregulating the PI3K/AKT/mTOR signaling pathway based on network pharmacology and experimental investigation
Source: Front Pharmacol. 2022 Nov 24;13:1054803. doi: 10.3389/fphar.2022.1054803 (PMC9729289; doi:10.3389/fphar.2022.1054803)
Supplement: Supplementary file 1 [file DataSheet2.DOCX]

Supplementary Material

# Supplementary Figures


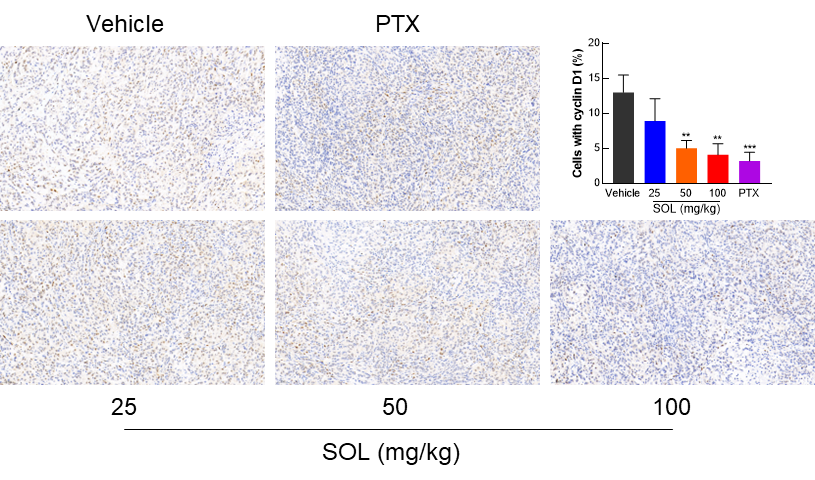


**Supplementary Figure 1 The expression of cyclin D1 in the tumor tissue of A549-bearing athymic nude mice was analyzed by the immunohistochemistry method.** Magnification: 200×, Scale bar: 50 µm. Bars in the histograms represent the mean ± SD in each group. Compared with the vehicle group, ** *P*＜0.01, *** *P*＜0.001.


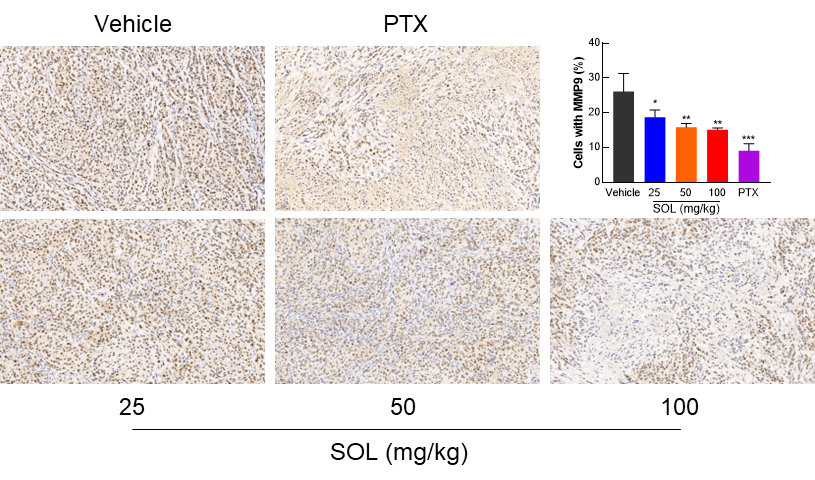


**Supplementary Figure 2 The expression of MMP9 in the tumor tissue of A549-bearing athymic nude mice were analyzed by the immunohistochemistry method.** Magnification: 200×, Scale bar: 50 µm. Bars in the histograms represent the mean ± SD in each group. Compared with the vehicle group, * *P*＜0.05, ** *P*＜0.01, *** *P*＜0.001.


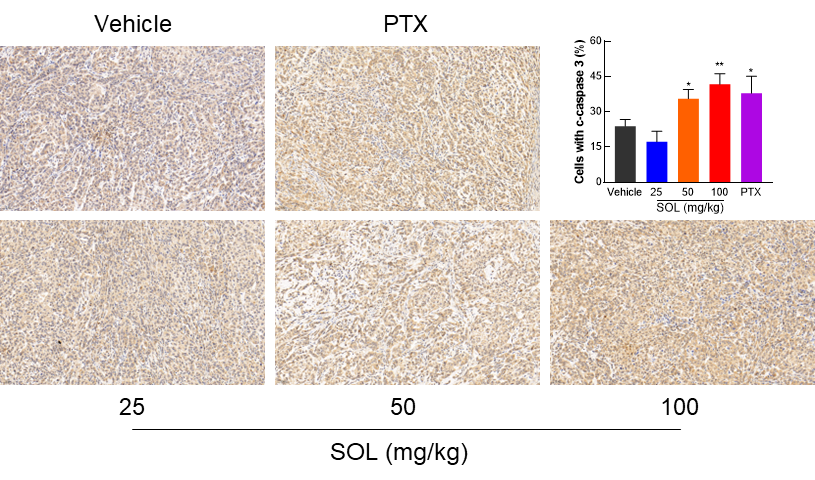


**Supplementary Figure 3 The expression of c-Caspase 3 in the tumor tissue of A549-bearing athymic nude mice were analyzed by the immunohistochemistry method.** Magnification: 200×, Scale bar: 50 µm. Bars in the histograms represent the mean ± SD in each group. Compared with the vehicle group, * *P*＜0.05, ** *P*＜0.01.


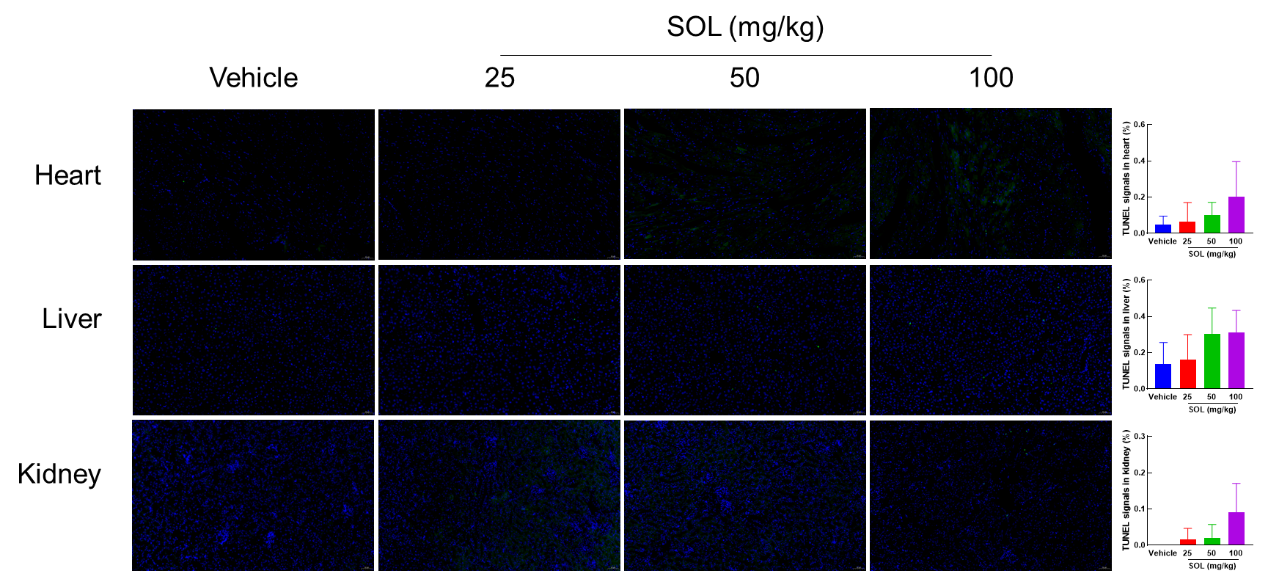


**Supplementary Figure 4 TUNEL staining on the heart, liver and kidney in A549-bearing athymic nude mice.** Magnification: 200×, Scale bar: 50 µm. Bars in the histograms represent the mean ± SD in each group.
