# Supplementary material for: Sanguisorba officinalis L. suppresses non-small cell lung cancer via downregulating the PI3K/AKT/mTOR signaling pathway based on network pharmacology and experimental investigation
Source: Front Pharmacol. 2022 Nov 24;13:1054803. doi: 10.3389/fphar.2022.1054803 (PMC9729289; doi:10.3389/fphar.2022.1054803)
Supplement: Supplementary file 2 [file DataSheet1.DOCX]

All raw data can be found in the link:

https://www.jianguoyun.com/p/DYOm6dcQ5uC_CRihu9cEIAA
